# Type 2 diabetes mellitus patients’ lived experience at a tertiary hospital in Ekiti State, Nigeria

**DOI:** 10.1038/s41598-022-12633-3

**Published:** 2022-05-19

**Authors:** Anthonia I. Okurumeh, Oluwaseyi A. Akpor, Olutoyin E. Okeya, Oghenerobor B. Akpor

**Affiliations:** 1 Department of Nursing Science, Afe Babalola University, PMB 5454, Ado-Ekiti, Ekiti Nigeria; 2 Department of Nursing Education and Primary Care, Faculty of Health, Social Care and Medicine, Edge Hill University, Ormskirk, Lancashire UK; 3 Department of Biological Sciences, Afe Babalola University, PMB 5454, Ado-Ekiti, Ekiti Nigeria

**Keywords:** Diseases, Health care

## Abstract

Diabetes mellitus is a complex and chronic metabolic disorder that is associated with multiple complications and disabilities. This contributes to increased mortality and poor quality of life among affected individuals. The study explored the lived experience of patients with type 2 diabetes mellitus at a Teaching Hospital in Ekiti State, Nigeria. A mixed method of qualitative and quantitative design was adopted. For the quantitative aspect, a convenience sampling technique was employed while the instrument used was an adapted questionnaire. For the qualitative aspect, focus-group discussion involving twenty-four participants was conducted, and the sample size was determined by data saturation. Qualitative data was analyzed using thematic transcription. Findings revealed that 55.6% of the participants were females while 63.5% had tertiary education. Majority (18 of 24) of the respondents experienced body weakness, frequent urination and excessive thirst when diagnosed of diabetes mellitus and later experienced occasional body weakness, burning sensation, tingling and numbness of the feet, fatigue, loss of libido, and occasional visual disturbance. Two-third of the participants reported being indifferent when they were informed about their condition. However, majority of the participants perceived that the cause of diabetes mellitus was heredity. More than two-third of the participants did not experience reduction in their normal daily activities but rather experienced occasional emotional disturbances, anxiety and challenges with self-management of diabetes and this was associated with maintaining a normo-glycemic state due to the financial implications of drugs and dietary modifications.

## Introduction

Diabetes mellitus (DM) is a complex and chronic metabolic disorder that is associated with multiple complications and disabilities. This contributes to increased mortality and poor quality of life among affected individuals^1^. Globally, the prevalence of diabetes is constantly increasing, thereby becoming a major global health challenge. The burden is higher in developing countries, such as Nigeria where more than 80% of the people live with Diabetes^2–4^. As a result of the high burden, diabetes mellitus is fast becoming an epidemic in some countries, especially developing countries with approximately 9.3% affected persons worldwide, the figure is expected to increase to 10.2% by the year 2030^2^.

Type 2 Diabetes Mellitus is a lifelong disease that requires continuous lifestyle control and modification^5^. However, management of type 2 diabetes presents numerous challenges to the attainment of optimal health care, many of which are inherent in the fears and perceptions of PLWD^6^. The focus of diabetes management places the responsibility of maintaining adequate glycemic control on the patients. Patients find this difficult to maintained, especially young adults who may be struggling to cope with other physiological and sociological stresses in their lives^7^. In other words, patients’ ability to achieve normal glycemic control is governed by not only their experiences regarding the initial diagnosis, but also their perceptions of the long-term difficulties of living with the disease^8^. Current understanding of such attitudes and beliefs is rudimentary; but notwithstanding, this is vital if patients are to achieve good glycemic control thereby curtailing diabetes-related complications and promote health care guideline implementation^5^.

Literature shows that the incidence of diabetes mellitus is increasing, but its related complications are preventable^8^. Some patients lack the required knowledge and skills to manage and control their conditions and this eventually may lead to complications. Thus, affecting their day-to-day activities which can consequently reduce their quality of life and contribute to negative physical, psychological and social experiences^5^. Type 2 diabetes complications can have tremendous impact on the mental health of patients. Moreso, prevalence of depression was identified to be approximately higher in patients with type 2 diabetes mellitus which is also associated with suicidal attempt with impaired health-related quality of life^9,10^.

It is opined that psychosocial problems are common among people living with diabetes (PLWD) and this often results in serious negative experiences, negative impact on patient's well-being and social lives if left un-addressed. Cognitive, emotional, behavioral and social factors are required in the psychosocial treatment interventions as these would help to overcome the psychological barriers that are associated with adherence and self-care for PLWD^11^.

Literature search shows that most studies conducted in Nigeria on diabetes mellitus are either on knowledge^12^, prevalence and current trends^13–15^, coping with DM^16^ among others. No study was seen to have combined the experiences of PLWD in its totality such that it will include vital concepts like knowledge, medication adherence, coping strategy, experiences during and after diagnosis, self-monitoring blood glucose, dietary management and so on. According to previous studies^17–19^, DM was noted to be responsible for over 50% of all lower extremity amputations, this finding is also similar to the clinical statistics of the study setting. It was further observed that most studies conducted on the lived experience of patients with type 2 diabetes mellitus adopted either the qualitative or quantitative research strategies. These studies were not conducted in Nigeria hence, the choice of a mixed method research. Therefore, the study explored the lived experience of patients with Type 2 diabetes mellitus attending an endocrinology clinic at a Teaching Hospital in Ekiti State, Nigeria.

## Methodology

### Research design and setting

This study used a concurrent mixed method research design, this involved quantitative and qualitative data collection at the same time or in parallel. This study was carried out at the endocrinology clinic of a Teaching Hospital in Ekiti State, Nigeria.

The facility was purposively selected due to high influx of diabetic patients. The facility receives an average of 70 clients per clinic, as revealed by the clinic register. Clinic runs once a week with a population of about 70 clients on weekly basis, resulting in about 140 clients in 2 weeks and 280 patients a month.

The target population for this study were PLWD attending endocrinology clinic at a Federal Teaching Hospital in Ado-Ekiti, the capital of Ekiti State. The inclusion criteria comprised of patients attending endocrinology clinic during the time of data collection and the willingness to participate in the study.

### Sample size and technique

The sample size for the quantitative aspect of the study was determined as follows:

$$n=\frac{N}{1+N(e)}2$$

where: n = desired sample size. N = Population size = 184. e = sampling error (0.05 as acceptable error).

The population size of 184 was obtained based on clinic record of the endocrinology unit of the Teaching Hospital as at January 2020. Based on the sample size calculation, a total of 139 respondents was calculated after factoring 10% attrition. Of the total number of 139 questionnaires administered, only 126 of them were retrieved.

For the qualitative aspect of the study, sample size for the study was determined by data saturation. While paying attention to the scope of the study, quality of data gotten, nature of the research topic, the study design and the presence of shadow data. This was done to ensure that there are no new or lost information. Six participants were recruited per clinic for 1 month making a total number of 24 participants.

### Sampling technique

Purposive Sampling technique was used to select diabetic patients from the endocrinology clinic. Purposive sampling technique was used because of the peculiarity of patients’ health status, to focus on the particular characteristics of the population of interest and also to answer the research questions.

The research instrument used for the quantitative aspect of the study was a structured adapted questionnaire that was designed based on information adapted from several similar studies^12,20,21^, as well as from relevant literature search. Adjustments were made to meet the objectives of the study. The questionnaire consisted of five sections; section A: which focused on the demographic characteristics (such as age, gender, ethnicity, religion, marital status, level of educational, occupation and average monthly income). Section B consisted of clinical characteristics and biophysical profile such as duration of diabetes, family history of diabetes, BMI, height, weight, RBS, blood pressure, comorbidity, pattern of anti-diabetic drugs, present of physical disability). Section C assessed the knowledge of diabetic patients on diabetes mellitus which was adapted from relevant literature search and modified to achieve the study’s objectives^12,21,22^. The question consisted of 40 items, with the correct item scored ‘1’ while incorrect item ‘0’. Section D assessed the level of adherence of patient to diabetic treatment regimen using a validated questionnaire, Medication Adherence Questionnaire (MCQ) that was adapted from Ahmad et al.^16^. These questions assessed intentional and unintentional non adherence of patient to diabetic treatment. A 4-point of Likert scale was allotted to each question Section E assessed diabetes coping strategies used by the patients, the question also consists of 14 items measured on 4 Likert scale from 0 to 3. The questionnaire was translated to the local language before being administered to the patients who could not comprehend English language and transcribed back to English language for result interpretation and analysis (Supplementary Information).

### Sampling technique

For the qualitative aspect, purposive sampling technique was used to select six patients from endocrinology clinic on weekly basis for 4 weeks. Data was gathered through the use of focus-group discussion. To guide the focus-group discussion, a guide was developed to explore the lived experience of diabetic patients while questionnaire was used to obtained demographic profile of the participants. The discussion was conducted in one of the consulting rooms at the endocrinology clinic with only the researcher and the participant so as to ensure privacy. The guide that was used for this study consisted of two sections; the first section was the introductory aspect, the second section was open-ended questions to elicit information on their age at initial diagnosis of DM, their experiences during and after the diagnosis, experience with managing and coping with diabetes like self-monitoring of blood glucose, dietary modifications, medication adherence and how the experiences affects their daily activities (diet, work, adherence to drug, relationship with spouse).

### Data analysis

The completed questionnaire collected was coded and analysed using Statistical Package for Social Sciences (SPSS) version 25. Descriptive statistics was used to summarized the data and provide clear description of the data from sample using frequency distribution tables, and percentages. Chi-square test was used for inferential statistics at probability level of 0.05.

The data collected with audio recorder from the Focus Group Discussion was transcribed verbatim and analyzed with the aid of Atlas.ti, using Thematic and Content Analysis, and was reported concurrently with the quantitative results.

### Ethical approval

Before the commencement of the study, approval for the study was obtained from the Research and Ethics Committee of the Federal Teaching Hospital, Ido-Ekiti, Ekiti State, Nigeria. In addition, detailed explanation was provided to all eligible respondents and informed consent obtained before their participations. All respondents were informed of the purpose of the study, assured of no harm, strict confidentiality and the right to self-determination was followed by providing the participants with the rights to voluntarily consent or decline to participate, and to withdraw at any time without penalty. Participants were also informed about the purpose and the procedure that will be used to collect the data and assured that there will be no potential risk or cost involved. Privacy and anonymity were ensured as the name of the participants or any form of identity will not be required in the questionnaire and information supplied by the participants will not be traced back to them.

All methods were performed in accordance with the relevant guidelines and regulations by including a statement.

## Results

### Demographic, Socio-economic status, biophysical and medical history of respondents

The socio-demographic characteristics of the respondents revealed that majority 36 (28.6%) of the respondents were between ages 60–69 years while 23.8% were between the ages of 40–49 years. The mean age was 57.8 ± 12.9 years, more than half 70 (55.6%) of the respondents were females, nearly all 93 (73.8%) were Yoruba, 85 (67.5%) were married while 30 (23.8) were widows. Above three-fourth 97 (77.0%) of the respondents were Christians (Table 1). The result of the socio-economic status of the respondents revealed that about two-third 80 (63.5%) of the respondents had tertiary level of education, majority 41 (32.5%) were civil servants followed by retiree with percentage of 36 (28.0%). Nearly half 65 (49.2%) earned about 70,000 naira and above on monthly basis. Majority 105 (83.3%) were neither smoking nor take alcohol (Table 1).

**Table 1:** Socio-demographic characteristics of the respondents (N = 126).

Characteristics	Variables	Frequency	%
Age (years)	< 30	2	1.6
	30–39	7	5.6
	40–49	30	23.8
	50–59	23	18.3
	60–69	36	28.6
	≥ 70	28	22.2
Gender	Male	56	44.4
	Female	70	55.6
Ethnicity	Yoruba	93	73.8
	Ibo	21	16.7
	Hausa	5	4.0
	Others	7	5.6
Marital status	Married	85	67.5
	Divorced	5	4.0
	Widowed	30	23.8
	Separated	6	4.8
Religion	Christianity	97	77.0
	Islam	28	22.2
	Traditional	1	0.8
Level of education	No formal education	7	5.6
	Primary education	5	4.0
	Secondary education	34	27.0
	Tertiary education	80	63.5
Occupation	Trading/business	21	16.7
	Skilled artisan	5	4.0
	Student	3	2.4
	Unemployed	5	4.0
	Self employed	15	11.9
	Civil servant	41	32.5
	Retiree	36	28.6
Monthly income (Naira)	< 10,000	10	7.9
	11,000–30,000	11	8.7
	31,000–50,000	19	15.1
	51,000–70,000	24	19.0
	≥ 70,000	62	49.2
Cigarette smoking	Yes	21	16.7
	No	105	83.3
Alcohol intake	Yes	24	19.0
	No	102	81.0

The medical history of patients with diabetes revealed that more than two-third 85 (67.5%) of the respondents had family history of type 2 diabetes mellitus with duration of diagnosis less than 5 years 54 (42.9%). Slightly above two-third of the respondents had hypertension as comorbidity, majority 84 (66.7%) takes 2–3 different drugs with combination of two or more tablets. Majority 86 (68.3%) of the respondents has no physical disability while 28 (22.2%) already had amputation of their limbs (Table 2). The biophysical profile of the respondents showed that majority 61 (48.4%) of the respondents had weight of 161–180 cm, weighed 71-70 kg 52 (41.3) with BMI of 25–29.9 (overweight). More than half 67 (53.2%) had normo-glyceamic (RBS) value of 80-160 mm/l and borderline blood pressure of 140/90mmhg-150/100 mmhg (Table 2).

**Table 2:** Medical history and biophysical profile of the respondents (N = 126).

History/profile	Variables	Frequency	%
**Medical histo**ry			
Family history of diabetes	Yes	85	67.5
	No	41	32.5
Duration of diabetes	< 5 years	54	42.9
	5 – 9 years	36	28.6
	10–14 years	22	17.5
	15–20 years	10	7.9
	> 20 years	4	3.2
Co-morbidity	Hypertension	86	68.3
	No associated diseases	40	31.7
Number of medications taken	1	18	14.3
	2	48	38.1
	3	36	28.6
	4	14	11.1
	5	10	7.9
Pattern of anti-diabetes	Tablets only	25	19.8
	Injection only	18	14.3
	Both tablets and injection	36	28.6
	Combination of two or more Tablets	47	37.3
Physical disability	No physical disability	86	68.3
	Amputation	28	22.2
	Blindness	12	9.5
**Biophysical profile**			
Height	121–140	18	14.3
	141–160	47	37.3
	161–180	61	48.4
Weight	< 50 kg	12	9.5
	51–70 kg	45	35.7
	71–90 kg	52	41.3
	91–110 kg	17	13.5
BMI	Normal (18.5–24.9 kg/m^2^)	28	22.2
	Overweight (25–29.9 kg/m^2^)	68	54.0
	Obesity (> 30 kg/m^2^)	30	23.8
RBS	Hyperglyceamic (above 160 mm/l)	59	46.8
	Normoglyceamic (80–160 mm/l)	67	53.2
Blood pressure	< 90/60	5	4.0
	90/60–130/90	42	33.3
	140/100–150/100	53	42.1
	> 150/100	26	20.6

### Knowledge, coping strategies and adherence of respondents

Generally, most of the study respondents had good knowledge (73%) of the disease condition. Only 24.5 and 1.6% of them had moderate and poor knowledge of the disease condition, respectively. The majority of respondents with good knowledge were age 60–69 years (34.8%), female (65.5%), Yoruba (69.6%), married (66.3%), Christians (72.8%) and were educated to tertiary level (77%). Age (X^2^ = 621.89, p ≤ 0.001), gender (X^2^ = 13.64, p ≤ 0.001), marital status (X^2^ = 15.74, p = 0.01) and highest educational level (X^2^ = 115.17, p ≤ 0.001) were observed to be significantly associated with the respondents’ knowledge of the disease condition. Ethnicity and religion were not observed to be significantly associated with the respondents’ knowledge of the disease (Table 3).

**Table 3:** Relationship between socio-demographic characteristics and knowledge of diabetes mellitus.

	Good	Moderate	Poor	Total	X^2^	p
**Age (years)**						
< 30	0 (0.0)	1 (3.1)	1 (50.0)	2 (1.6)	621.89	< 0.001
30–39	4 (4.3)	3 (9.3)	0 (0.0)	7 (5.6)		
40–49	12 (13.0)	18 (56.3)	0 (0.0)	30 (23.8)		
50–59	19 (20.7)	3 (9.3)	1 (5.0)	23 (18.3)		
60–69	32 (34.8)	4 (12.4)	0 (0.0)	36 (28.6)		
≥ 70	25 (27.2)	3 (9.3)	0 (0.0)	28 (22.2)		
**Gender**						
Male	32 (34.8)	22 (68.8)	2 (100)	56 (44.4)	13.635	< 0.001
Female	60 (65.2)	10 (31.2)	0 (0.0)	70 (55.6)		
**Ethnicity**						
Yoruba	64 (69.6)	27 (84.4)	2 (100)	93 (73.8)	2.734	0.746
Ibo	18 (19.6)	3 (9.3)	0 (0.0)	21 (16.7)		
Hausa	4 (4.3)	1 (3.1)	0 (0.0)	5 (3.9)		
Others	6 (6.5)	1 (3.1)	0 (0.0)	7 (5.6)		
**Marital status**						
Married	57 (66.3)	26 (68.8)	2 (100)	85 (67.4)	15.737	0.01
Divorced	4 (4.3)	1 (3.1)	0 (0.0)	5 (1.6)		
Widowed	29 (27.2)	1 (15.6)	0 (0.0)	30 (23.8)		
Separated	2 (2.2)	4 (12.3)	0 (0.0)	6 (4.8)		
**Religion**						
Christianity	67 (72.8)	28 (87.5)	2 (100)	97 (76.9)	3.597	0.463
Islam	24 (26.1)	4 (12.5)	0 (0.0)	28 (22.2)		
Traditional	1 (1.1)	0 (0.0)	0 (0.0)	1 (0.7)		
**Level of education**						
No formal education	0 (0.0)	5 (15.6)	2 (100.)	7 (5.6)	115.167	< 0.001
Primary education	0 (0.0)	5 (15.6)	0 (0.0)	5 (5.6)		
Secondary education	12 (13.0)	22 (68.8)	0 (0.0)	34 (26.9)		
Tertiary education	80 (77.0)	0 (0.0)	0 (0.0)	80 (63.5)		
Knowledge level	92 (73)	32 (25.4)	2 (1.6)			

Values in parenthesis indicate % while X^2^ and p represent chi-square and probability values, respectively.

With respect to coping strategies, almost half of the respondents (50.8%) had good coping strategies while 41.3 and 7.9% of them had moderate and poor coping strategies, respectively. Out of the total respondents that had good coping strategies, 39.1% were aged 60–69 years, 84% were females, 75% were Yorubas, 78.1% were married, Christians and educated up to the tertiary level. Out of the socio-demographic characteristics investigated, age ((X^2^ = 643.72, p ≤ 0.001), gender (X^2^ = 46.97, p ≤ 0.001), marital status (X^2^ = 13.10, p = 0.041) and highest educational level (X^2^ = 79.92, p ≤ 0.001) were showed to be significantly associated with the respondents’ coping strategies for diabetes mellitus. No significant association was observed between ethnicity and religion with coping strategies for the disease condition (Table 4).

**Table 4:** Relationship between socio-demographic characteristics and coping strategies of diabetes mellitus.

	Good	Moderate	Poor	Total	X^2^	p
**Age (years)**						
< 30	0 (0.0)	0 (0.0)	2 (20.0)	2 (1.6)	43.71	< 0.001
30–39	1 (1.6)	2 (3.8)	3 (30.0)	7 (5.6)		
40–49	12 (18.8)	17 (32.7)	1 (10.0)	30 (23.8)		
50–59	10 (15.6)	12 (23.1)	1 (10.0)	23 (18.3)		
60–69	25 (39.1)	13 (25.0)	1 (10.0)	36 (28.6)		
≥ 70	16 (25.0)	8 (12.5)	2 (20.0)	26 (20.6)		
**Gender**						
Male	10 (15.6)	36 (69.2)	10 (100)	56 (44.4)	46.97	< 0.001
Female	54 (84.4)	16 (30.8)	0 (0.0)	70 (55.6)		
**Ethnicity**						
Yoruba	48 (75.0)	37 (71.2)	7 (70.0)	93 (73.8)	1.97	0.922
Ibo	11 (17.2)	8 (15.4)	2 (20.0)	21 (16.7)		
Hausa	3 (4.7)	3 (5.7)	1 (10.0)	5 (3.9)		
Others	2 (3.1)	4 (7.7)	1 (10.0)	7 (5.6)		
**Marital status**						
Married	50 (78.1)	28 (67.3)	7 (70.0)	85 (67.4)	13.10	0.041
Divorced	3 (4.7)	1 (1.9)	1 (10.0)	5 (1.6)		
Widowed	10 (15.6)	19 (36.5)	1 (10.0)	30 (23.8)		
Separated	1 (1.6)	4 (7.7)	1 (10.0)	6 (4.8)		
**Religion**						
Christianity	50 (78.1)	41 (78.8)	6 (60.0)	97 (76.9)	2.93	0.569
Islam	13 (20.3)	11 (11.2)	4 (40.0)	28 (22.2)		
Traditional	1 (1.6)	0 (0.0)	0 (0.0)	1 (0.7)		
**Level of education**						
No formal education	1 (1.6)	0 (0.0)	6 (60.0)	7 (5.6)	79.92	< 0.001
Primary education	2 (3.2)	1 (1.9)	2 (20.0)	5 (5.6)		
Secondary education	11 (17.2)	21 (40.4)	2 (20.0)	34 (26.9)		
Tertiary education	50 (78.1)	30 (57.7)	0 (0.0)	80 (63.5)		
Coping level	64 (50.8)	52 (41.3)	10 (7.9)			

In the case of medication adherence, a vast majority of the respondents (75.4%) were observed to adhere to treatment medication. Highest education level, (X^2^ = 16.97, p ≤ 0.001), occupation (X^2^ = 16.52, p = 0.011) and monthly income (X^2^ = 10.03, p = 0.01) showed significant association with medication adherence among the respondents (Table 5).

**Table 5:** Relationship of socio-economic status of respondents with medication adherence strategies of diabetes mellitus.

Level of education	Adherence	Non-Adherence	Total	X^2^	p
No formal education	1 (1.1)	06 (19.4)	7 (5.5)	16.972	< 0.001
Primary education	3 (3.2)	02 (6.5)	5 (4.0)		
Secondary education	25 (26.3)	09 (29.0)	34 (27.0)		
Tertiary education	66 (69.5)	14 (45.2)	80 (63.5)		
**Occupation**					
Trading/business	16 (16.8)	5 (16.1)	21 (16.7)		
Skilled artisan	3 (3.3)	2 (6.4)	5 (3.9)	16.523	0.011
Student	2 (2.2)	1 (3.2)	3 (2.4)		
Unemployed	4 (4.4)	1 (3.2)	5 (3.9)		
Self employed	11 (11.6)	4 (12.8)	15 (11.9)		
Civil servant	38 (40.0)	3 (9.6)	41 (32.5)		
Retiree	19 (20.0)	17 (54.8)	36 (28.6)		
**Monthly income (Naira)**					
< 10,000	7 (7.4)	3 (9.7)	10 (7.9)	10.032	0.039
11,000–30,000	6 (6.3)	5 (16.1)	11 (8.7)		
31,000–50,000	15 (15.8)	4 (12.9)	19 (15.1)		
51,000–70,000	14 (14.7)	10 (32.3)	24 (19.1)		
≥ 70,000	53 (55.8)	9 (29.0)	62 (49.2)		
Medical adherence level	95 (75.4)	31 (24.6)			

Values in parenthesis indicate % while X^2^ and p represent chi-square and probability values, respectively.

Content analysis conducted on the data from the focus group discussion led to the development of four themes and fifteen subthemes emerged (Table 6).

**Table 6:** Main themes and subthemes generated from data.

Theme	Main theme	Sub-themes	Respondents (n = 24)
1	Respondents’ experiences during the diagnosis	Symptoms experience during the diagnosis	18 (75.0%)
		Acceptance and EMOTIONAL experience when diagnosed with diabetes	16 (66.7%)
		Perception about causes of diabetes	22 (91.7%)
		Motivation to control diabetes	18 (75.0%)
2	Respondents’ experience after diagnosis	Means of taking care of diabetes	22 (96.7%)
		Diabetes limits life chance	18 (75.0%)
		Physical symptoms experience with diabetes	22 (91.7%)
		Emotional experience with diabetes	18 (75.0%)
		Social problems associated with diabetes	12 (50%)
3	Experience with managing and coping with diabetes	Measures to manage diabetes and keep blood sugar within normal range	24 (100%)
		Experience with the use of diabetic drugs and checking of blood sugar	22 (96.7%)
		Approaches to prevention of complications	21 (87.5%)
4	Experience with self-management of diabetes	Experience with daily diabetes self-care	18 (75%)
		Experience with managing blood sugar	24 (100)
		Experience with financial cost of managing diabetes	20 (83.3%)

### Experience during the diagnosis stage

Majority (18 of 24) of the respondents stated that they experience general body weakness prior the diagnosis of diabetes, while other responded that they urinate 4–5 times before the day break couple with excessive thirst. However, other reported that they have no significant symptoms prior diagnosis aside occasional malaria symptoms.

A respondent stated.

*“I experienced occasional malaria symptoms which progressed to generalized body weakness and profuse sweating thinking it was resistance malaria before I was diagnosed of diabetes in the hospital. Sometimes, I used to drink a lot of water and urinate many times at night”. (FGDS 1, 60-year-old Retiree).*

Two-third (16 of 24) of the respondents reported being indifferent when they were informed about their condition. They accepted it initially with light heart but later became anxious about the complications after being told about the health implications and financial cost of managing diabetes mellitus. The participants later accepted the condition because they have to live with the condition, take some precautionary measures and use their medications regularly. A participant reported that;

*‘Initially when the diagnosis was made I was indifferent because I don’t know what it takes to manage the condition but when I was counseled on certain lifestyle modifications and possible complications that may …………………………………………, I later became sad but late accepted my fate, I totally accepted the disease, not being pessimistic about it apart from the fact that I have background history of the disease, I tried as much as possible to prevent it but now that it has come I have to move on with life’ (FGDS 2, 62 year old Businessman).*

Majority 22(91.7%) of the respondents stated that they could not say precisely what could have been responsible for their condition but they belief too much sugar and carbohydrate can cause diabetes. Most importantly, gene and heredity can as well cause type 2 diabetes mellitus. A participant stated that;

*‘I don’t know what caused my diabetes but the diagnosis was made when I was sick thinking it was malaria fever until I got to the hospital…….., I think it is hereditary because some of my family members have it’ (FGD 3. 70-year-old Retiree).*

Three-fourth, 18 (75%) of the respondents reported that they were motivated by their family member including spouses and friends to control their diet and do certain exercise because they don’t want to die prematurely. One of the participants expressed the need to control her blood sugar;

*¹I need to control my blood sugar through diet and regular intake of prescribed drugs, though it wasn’t easy but with support and motivation received from my children and wife including my friends I find it easy to follow recommended instruction’. (FGD 4, 74-year-old female Dependant).*

### Experience after the diagnosis

Majority 22 (96.7%) stated that they learn to take care of their disease through health talk and counseling by the Doctors and Nurses on lifestyle modification, dietary modifications and regular exercise. One of the participants reported that.

*‘I learnt a lot from the medical personnel especially Nurses, I followed strictly dietary education, adherence to drug and regular follow up visit which has reduced my tension about the diseases’. (FGD 2, 68-year-old Retiree).*

More than three- fourth (18 of 24) of the respondents reported that disease did not reduce their normal daily activities nor reduced their life chances except for the fact that they followed strict diet at all times and also check their blood sugar regularly. A participant in focus group 4 stated that;

*‘Diabetes has not really affected me in any way, I am fine and I’m carrying out my normal daily activities. Nothing has really changed with me except for the food restriction. Anyway, I’m coping well’ (FGD 4, 48-year-old civil servant).*

Nearly all (22 of 24) the participants experienced symptoms of tiredness, generalized body weakness, occasional dizziness, frequent urination and excessive thirst as well as burning sensation, tingling and numbness in their hands and feet, fatigue, loss of libido, and occasional visual disturbance. A participant in focus group discussion 3 stated that;

*‘I always have numbness and pin sensation in my hand and feet usually at night, I experience fatigue a lot and I urinate too much most especially in the night about 5–6 times before morning. This often affect my sleep and reduce my activities the following day’ (FGD 3, 52-year-old Trader*).

About three-fourth, 18 (75.0%) of the respondents stated that they were worried about high blood sugar level, occasional weakness as well as other symptoms experienced when their blood sugar level dropped below the normal value, most especially the complications observed in other patients. A participant who expressed emotional problem said;

*‘I am always disturbed whenever I visit clinic for follow up and I see other patients with complications especially those that have lost their sight and have one of their limbs amputated. Infact, I always have negative emotional feelings anytime I came back from clinic’. (48-year-old Civil Servant).*

Half (12 of 24) of the respondents reported that they experienced social isolation when in social gathering or family functions because they could not eat what others were eating. They reported feeling restricted going to social gathering but nonetheless, they still manage to go with caution and consciousness of their health condition. Half of the respondents also reported that they do not honour invitation to social gathering where meal will be involved.

*‘I rarely go for social gathering because my social life is completely reset, I couldn’t do much with my peers, I deliberately distance myself from my colleagues to avoid embarrassment and stigmatization. Moreso, I am too young to disclose my condition to anyone. (26-year-old Graduate unemployed).*

### Experience with managing and coping with diabetes

All the respondents 24 (100%) responded that they managed their condition by taking their prescribed drugs regularly, adhering strictly to the instruction of medical personnel, modification and regulation of diet especially the reduction of carbohydrate and sugary drinks, engaging in weight reduction measures to keep weight in check, regular checking of blood sugar, regular exercise and checkup /follow up visits at the hospital. One of the respondents stated that;

*‘I make sure I manage my disease well to prevent complications, I adhere to dietary instruction of nurses, use my drug regularly and check my blood sugar twice a day usually in the morning and evening, I also do exercise before going to shop to reduce my weight. I am really coping well with the disease by strictly following the instructions associated with managing diabetes mellitus’. (FGD 2, 53-year-old Trader).*

Nearly all 22 (96.7%) stated that using drugs is frustrating and tiring, using drugs in the public attract some stigmatization that could be so disheartening. More so, they are always afraid anytime they want to check their blood sugar, thinking that the value will be elevated especially when have not followed dietary instruction. One of the participants reported that;

*It is not easy to be on drug, it is money consuming, and sometimes I used all the money I have on drugs. I get fed up most time especially when the drugs are almost finished. …………….. More so, I usually afraid of checking my blood sugar particularly when I bridge dietary instructions, in that wise I used to take my drug before checking the blood sugar’. (FGD2, 62-year-old business man.*

More than two-third (21 of 24) of the participants reported that they prevent complications associated with diabetes mellitus by using their drugs regularly, following dietary instructions, good compliance and adherence to medication regimen, regular exercises, skin care such as paying more attention to toes and feet and mouth care. One of the participants stated that;

*‘I am afraid of complications; I don’t pray to experience it at all. Therefore, I take my drugs regularly, I ensure personal hygiene are taken care of, most especially my feet, I don’t use blade to cut my nails and I always conscious of my shoes, I use loose and soft shoes and I take my drug regularly’. (FGD 3, 70-year-old Retiree).*

### Experience with self-management of diabetes

Majority 18 (75.0%) of the respondents reported that the biggest struggles they encounter with self-management of diabetes mellitus are daily checking of blood sugar and self-injection with insulin including restriction to their favorite meal. A participant stated that;

*‘I am not always happy checking my blood sugar and injecting myself. Though I have to learn it and carry it out when there is need for it. However, it is a very difficult task to carry out on daily basis, I have no choice, if I want to be healthy’. (FGD 1,62-Year-old Retiree).*

All (24 of 24) the participants have experienced low blood sugar at one time or the other with great fear and anxiety whether they will survive it or not, however, the participants responded that they managed low blood sugar by taken high carbohydrate diets such as sweetened drinks but with caution. One of the participants stated that;

*‘I always afraid of low blood sugar because I have experience it on several occasion. I go about with cube sugar in case of sudden low blood sugar. I also take cola drink whenever I have low blood sugar which I do with caution to avoid elevated blood sugar level’. (FDG 4, 48-year-old Civil servant).*

Nearly all (20 of 24) of the participants stated that they experience some financial constraints in purchasing the required glucose monitoring equipment, insulin injections and drugs. In addition, recommended dietary intake is also expensive unlike normal meals. One of the participants share his financial experience with managing diabetes.

*‘I experience financial constraints in getting all I need to manage my condition, I used strip every day to monitor my blood sugar, I take drug every day, I could not eat as much as I want and what I wanted to eat. All these are daily struggles I faced in taking care of my condition every day. It is not easy!’. (FGD 3, 68-year-old Retiree).*

## Discussion

The findings from this study showed that majority of the respondents were between the ages 60–69 years followed by ages 40–49 years with the mean age of 57.8 ± 12.9, more than half (56%) were females. The result is in tandem with Bukhsh et al.^23^ who reported mean age of 57.8 ± 10.9 years and majority (67%) of the respondents were married. However, in contrast to the findings from the study. Roy et al.^24^ also reported that there are more males than females among PLWD. However, male gender was associated with higher incidence of other co-morbidity such as diabetes mellitus, congestive cardiac failure and peripheral arterial disease which is contrary to the result of the study. It is indicated that middle-aged and older adults of 45–64 years are still at the highest risk of developing type 2 diabetes mellitus^20^.

Participants medical history with diabetes shows that more than two-third had family history of type 2 diabetes mellitus with duration of diagnosis less than 5 years. Slightly above two-thirds also had hypertension as a comorbidity. The most constant factor for developing diabetes is family history of diabetes, especially from the first-degree family members. There is high tendency for people with family history of diabetes to develop diabetes later in life, more so, cardiovascular health conditions like high blood pressure, low level of HDL, and other forms of heart condition are greatly associated with type 2 diabetes mellitus^25^.

Findings from the study showed the biophysical profiles of the respondents. Majority of the respondents had height and weight of 161–180 cm and 71–70 kg with BMI of 25–29.9 (overweight). More than half had normo-glyceamic (RBS) value of 80–160 mm/l and borderline blood pressure of 140/90–150/100 mmhg. Bukhsh et al.^23^, reported that most diabetic patients were obese and overweight with poor glycemic control. Similar observation has been reported by earlier investigators^21^.

About three-fourth of the respondents had good knowledge of type 2 diabetes mellitus while only one-fourth had moderate knowledge. This observation is in contrast to the findings from the study conducted by Khaldon et al.^26^ where it was reported that the overall knowledge of diabetic patients was very low with higher knowledge level among males. Razaleen and Nazirah^27^, stated that sufficient knowledge about diabetes is the key to diabetes care. Therefore, knowledge of care and sufficient health education is vital in molding patient attitude and believe in managing DM. Moreover, knowledge of DM is important in order to detect the present of related symptoms and to help avoid the practice of risky lifestyle^28^. In other words, inadequate knowledge is one of the main reasons for poor self-care and glycemic control^29^. However, Chirvala et al.^28^ reported poor knowledge about diabetes and its associated complications among patients. Furthermore, knowledge has direct effect on patients’ attitude and experiences, therefore patient with poor knowledge about such as its causes, types, correct nutrition, suitable activities and exercise treatment regimen may experience negative quality of life^30^.

Good adherence to diabetes management among the participants was observed in this study. According to a Malaysian study, more than half of their participants showed non-adherence to their medications^16^. Mualla et al.^8^ also stated that adherence to medication regimen and dietary recommendations as well as the development of self-management skills are critical to effective control and management of diabetes mellitus. However, adherence to medication is influenced by several factors, such as lack of information, complexity of regimen, concomitant diseases, perceptions of benefits, cost, medication side effects and emotional wellbeing of the patient^16^. Non adherence to medication contributes significantly to failure to achieve optimal therapeutic outcomes. It is also found that education may increase patients' ability to adopt and adhere to complex new diabetes treatments which often require careful patient self-management on a daily basis. For example, patients must monitor their blood glucose levels, balance insulin injection doses with food intake and physical activity, and consult regularly with health care providers^21^. Challenges of treatment adherence has been linked to psychological barrier associated with fear of needle and injection, insulin initiation, hypoglycemic late complication and obsessive behaviour of overdosing^24^.

Also, findings revealed that more than half of the respondents had good coping strategies with diabetes mellitus. It was reported that the highest level of emotional-focused coping among patients with diabetes mellitus can directly influence diabetes-related self-care activities. This suggests that patient with high level of coping will be more likely to maintain optimal glycemic control. Therefore, coping mechanism have a significant impact on the quality of life and diabetes related self-care activities^31^.

Further findings also showed that majority of the respondents have experienced general body weakness prior to the diagnosis of diabetes, frequent and excessive thirst coupled with polyuria about 5–6 times in the night, occasional dizziness, and difficulty with breathing. Frequent urination, dry mouth and frequent unexplained infection. American Diabetes Association^25^ also reported that the common symptoms of diabetes mellitus are the 3 ps, which are polyuria, polydipsia, and polyphagia, other common symptoms include extreme fatigue, blurry vision, weight loss, tingling, pains, or numbness in the hands and feet.

In addition, majority of the respondents in the study were indifferent about their diagnosis initially, but later became anxious about the complications. They expressed some levels of fear and dissatisfaction after being told about the possible health and financial implications of managing diabetes mellitus. Participants later accepted their diagnosis since it is a lifelong condition by taking necessary precautionary measures and use their prescribed medications regularly. Mualla et al.^8^ stated that most PLWD experience feeling of shock, denial, excessive stress, sadness, grief and anxiety when they were first diagnosed. But some later came to term with it while others did not accept or adapt to the disease by adopting an indifferent attitude or simply ignore and reject their diagnoses and as such this may affect their quality of life.

It is hypothesized that the primary goal in the treatment and early diagnosis of patients with diabetes mellitus is quality of life (QoL), which consists of physical, cognitive, psychological and social components^32^. Different scales and tools, such as the Audit of Diabetes-Dependent Quality of Life (ADDQoL), Cost of Diabetes (COD), and SF-36 have been used by various studies to measure the quality of life of patients with diabetes mellitus. A study by Ribu et al.^33^ used the SF-36 and concluded that patients with diabetic foot ulcer reported significantly poorer HRQL than the general diabetes population, diabetic foot ulcer patients had much worse HRQL, especially in physical health. Foot ulcer patients were more often men living alone, and obesity was a problem in both the foot ulcer patients and among the general diabetes population^33^. Trikkalinou et al.^32^ also used the COD tool to measure the quality of life of PLWD and concluded that there was better quality of life among individuals with higher income. In conclusion however, there are misunderstanding regarding the context of QoL, HRQoL and diabetes specific QoL. Hence, it would be ideal if the same psychometric tools could be translated validated and used in a worldwide scale in order to explore differences in the populations and extract comparable results.

## Conclusion

The study revealed that majority of PLWD had good knowledge about the disease and have developed good coping strategies. Consequently, they had good adherence to diabetes self-management. There was significant relationship between socio-demographic characteristics and knowledge of type 2 diabetes mellitus. A significant relationship was also observed between socio-economic status and adherence with diabetes medication. In addition, majority of the patients experienced overwhelming symptom when being diagnosed of type 2 diabetes mellitus which include physical symptoms, emotional disturbance as well as psychological distress. The self-management efforts made by the participants were dietary and lifestyle modifications, regular exercise, medication adherence, and regular blood sugar monitoring and follow-up. Nearly all the participants had experienced financial constraint in the course of managing type 2 diabetes mellitus.

## Supplementary Information

Supplementary Information.
