# Supplementary material for: Type 2 diabetes mellitus patients’ lived experience at a tertiary hospital in Ekiti State, Nigeria
Source: Sci Rep. 2022 May 19;12:8481. doi: 10.1038/s41598-022-12633-3 (PMC9120021; doi:10.1038/s41598-022-12633-3)
Supplement: Supplementary file 1 — Supplementary Information. [file 41598_2022_12633_MOESM1_ESM.docx]

**STUDY QUESTIONNAIRE**

**SECTION A: SOCIO-DEMOGRAPHIC CHARACTERISTICS**

Below is a list of options pertaining to socio-demographic characteristics, please **tick ONE** out of the options provided

- Age - (a) <30 years ( b ) 30-39 years ( c ) 40-49 years (c) 50-59 years (d) 60-69 years (e) 70years and above
- Gender - (a)Male ( b ) Female
- Ethnicity - (a) Yoruba ( b ) Ibo ( c) Hausa (d)Others ………………………
- Marital status - (a) Married (b) Divorced (c) Widowed (d) Separated (e) Cohabiting
- Religion - (a) Christianity (b) Islam (c) Traditional (d) Others………………………
- Level of education - No formal education ( ) Primary education ( ) Secondary education ( ) Tertiary education ( )
- Occupation - (a)Trading / Business (b)Skilled artisan ( c) Student

(d) Unemployed (e ) Self employed ( f) Civil servant ( g ) Retiree (h ) Others

- Average monthly income – (a) <10,000 naira ( b ) 11,000-30,000 naira ( c ) 31,000-50,000 naira ( d ) 51,000-70,000naira (e) 70,000 naira and above.

**SECTION B: CLINICAL CHARACTERISTICS AND BIOPHYSICAL MEASUREMENT**

- Cigarette smoking - (a) Yes (b) No
- Alcohol intake - (a) Yes (b) No
- Duration of diabetes - (a) < 5 years (b) 5 – 9 years ( c ) 10-14 years

(d) 15-20 years ( e ) >20 years

- Family history of diabetes - (a) Yes (b) No
- Comorbidity (Other chronic conditions)- (a)Hypertension ( b ) Heart Failure ( c) No associated diseases (d) others…………………………………………………
- Number of medications taken - 1 ( ) 2 ( ) 3 ( ) 4 ( ) 5 ( )
- Pattern of anti diabetic drugs - (a) Tablets only ( b) Injection only

( c ) Both Tablets and injection ( d ) Combination of two or more Tablets

- Physical disability - (a)No physical disability ( b ) Amputation ( c ) Blindness
- Height ………………………………………………………….
- Weight…………………………………………………………..
- BMI (a)Normal ( b ) Overweight ( c ) Obesity
- RBS……………………………………………………………..
- Blood pressure…………………………………………………

**SECTION C**

**KNOWLEDGE OF DIABETES**

Below is a list of questions related to knowledge of diabetes, these questions are designed to assess your level of knowledge on diabetes, please kindly TICK ONE option

|  | **General knowledge of diabetes** | Yes | No |
| --- | --- | --- | --- |
| 1 | Diabetes is a condition of high blood sugar |  |  |
| 2 | Diabetes cannot be cured |  |  |
| 3 | Insulin is required for some diabetic patients |  |  |
| 4 | Fasting blood sugar of 210 is too high |  |  |
|  | **Knowledge on risk factors, symptoms and complication of diabetes** |  |  |
| 5 | Diabetes can be inherited |  |  |
| 6 | Anybody above 40 years can have diabetes |  |  |
| 7 | Obesity can cause diabetes |  |  |
|  | **Symptoms of diabetes are** |  |  |
| 8 | Diabetes causes constant feeling of thirst |  |  |
| 9 | Diabetes causes frequent urination |  |  |
| 10 | Diabetes causes weight loss |  |  |
| 11 | Diabetes causes blurred vision |  |  |
| 12 | Diabetes causes slow healing of cuts and wound |  |  |
| 13 | Diabetes causes tiredness and weakness |  |  |
|  | **Diabetes can lead to the following complications** |  |  |
| 14 | Decay limbs that require removal (amputation) |  |  |
| 15 | Eye problems |  |  |
| 16 | Kidney problems |  |  |
| 17 | High blood pressure |  |  |
| 18 | Heart attack |  |  |
| 19 | Loss of sensation in the arm and legs |  |  |
|  | **Knowledge on the treatment and management of diabetes** |  |  |
| 20 | Insulin injection are available for the control of diabetes |  |  |
| 21 | Tablets and capsules are available for the control of diabetes |  |  |
| 22 | Diabetic patient should carry sugar when they are out |  |  |
| 23 | Diabetic patient should exercise regularly |  |  |
| 24 | Diabetic patient should have good weight control |  |  |
| 25 | Diabetic patient should Go for regular eye check up |  |  |
| 26 | Diabetic patient should Have a low fat and high fiber diet |  |  |
| 27 | Diabetic patient should Take care of their toes and feet |  |  |
| 28 | Diabetic patient should Not consume alcohol |  |  |
| 29 | Diabetic patient should Not donate blood |  |  |
| 30 | Diabetic patient should Control the intake of fruit and vegetables |  |  |
| 31 | Diabetic patient should Not smoke |  |  |
| 32 | Diabetic patient should Wear tight shoes |  |  |
| 33 | Diabetic patient should Skip meals when busy |  |  |
| 34 | Diabetic patient should Test for blood glucose |  |  |
| 35 | Diabetic patient should Test for sugar in the urine |  |  |
| 36 | Diabetic patient should Go for counseling section |  |  |
| 37 | Diabetic patient should Go for regular medical check ups |  |  |
| 38 | Diabetic patient should take extra caution when cutting nails |  |  |

**SECTION D:**

**MEDICATION ADHERENCE**

Below is a list of questions related to medication adherence, these questions are designed to assess your level of adherence to diabetic drugs, please kindly TICK ONE option

|  |  | **Always (daily)** | **Often (1-5 times monthly)** | **Sometimes (1-4 times monthly )** | **Never** |
| --- | --- | --- | --- | --- | --- |
| 1 | How often do you forget to take your drugs? |  |  |  |  |
| 2 | How often do you decide not to take your drugs |  |  |  |  |
| 3 | How often do you miss taking your drugs because you feel better |  |  |  |  |
| 4 | How often do you decide to take less of your drugs |  |  |  |  |
| 5 | How often do you stop taking your drugs because you feel sick due to effect of the drugs |  |  |  |  |
| 6 | How often do you forget to bring along your drugs when you travel away from home |  |  |  |  |
| 7 | How often do you not take your drugs because you run out of it at home |  |  |  |  |

**SECTION E:**

**DIABETES COPING STRATEGIES**

Below is a list of questions related to diabetes coping strategies, these questions are designed to assess your coping strategies in managing your diabetes, please kindly **TICK ONE** option.

|  |  | **Never** | **Sometimes** | **Often** | **Always** |
| --- | --- | --- | --- | --- | --- |
| 1 | Planning meal in accordance with the system being taught by health care providers |  |  |  |  |
| 2 | Weighing and measuring food limiting the amount of food that contains a lot of sugar or fat |  |  |  |  |
| 3 | Limiting the amount of food that contains a lot of sugar |  |  |  |  |
| 4 | Taking amount of insulin prescribed including adjustment based on blood glucose level |  |  |  |  |
| 5 | Taking insulin at the right time usually before each meal |  |  |  |  |
| 6 | Alternating injection site to avoid building up lumps (lipohyperthrophy) |  |  |  |  |
| 7 | Measuring blood glucose before each meal |  |  |  |  |
| 8 | Exercising or participating in some form of physical activity |  |  |  |  |
| 9 | Adjusting the amount of insulin based on glucose level |  |  |  |  |
| 10 | Adjusting amount of insulin when ill |  |  |  |  |
| 11 | Detecting and responding to early sign of low blood sugar |  |  |  |  |
| 12 | Keeping appointment and check up date |  |  |  |  |
| 13 | Keeping a diary of the amount of insulin being taken |  |  |  |  |
| 14 | Keeping record of daily blood glucose level |  |  |  |  |

**INTERVIEW GUIDE ON LIVED EXPERIENCE OF DIABETIC PATIENT DURING DIAGNOSIS, AND AFTER BEING DIAGNOSED WITH TYPE 2 DIABTES MELLITUS**

**SECTION A: INRODUCTION**

- Tell me something about yourself
- work ,
- family and
- what you enjoy doing in your spare time

**SECTION B: LIVED EXPERIENCE OF DIABETIC PATIENTS**

**1. Experience during the Diagnostic Stage**

- What were your symptoms at diagnosis?
- What was your emotional feeling when being diagnosed with diabetes?
- What do you think would have being responsible for your diabetes?
- Did you accept initially that you have diabetes or reject it?
- What are some of your motivation for wanting to control your diabetes?

**2. Experience after the Diagnosis**

- How did you learn to take care of your diabetes?
- Did diabetes limit life chances and what can you say about your condition and your activities of daily living in line with your diabetes?
- What are the emotional problems you experience with your diabetes?
- What are the physical symptoms you experience with your diabetes?
- Are you comfortable going to social gathering?
- **Experience with Managing and Coping with Diabetes**
- How did you adjust your life to keep your blood sugar normal?
- What do you do to manage your diabetes?
- Has diabetes affected your daily routine activities?
- What approach do you take to prevent complications?
- What are the problems you encounter in the use of your drugs?
- What do you do in particular that helps you the most with your diabetes?
- Did you always take your drugs?
- What is your usual thought when you take your blood sugar?

**4 Experience on Self Monitoring Blood Glucose Practice**

- In your own opinion what is the preferred method of recording your blood glucose?
- Do you think self-monitoring of blood glucose useful for your diabetes management?
- What put you off from taking care of your diabetes?

**5 Experience on Dietary Management**

- In your own opinion what are the strategies to control your diet?
- How do you handle your daily meal?

**6 Experience with Diabetes Medication**

- What are your experiences with diabetic medications?
- What type of experience with diabetes medication usually reduces your adherence to treatment?
- Do you aware of other beliefs in people that influence your diabetes management?
- Have you heard of alternative medicine for diabetics?
- What would you like to suggest that improve diabetes management behaviour among other diabetic patients?

**7 Experience with Barrier and Factors for Success in Diabetes Self Management**

- What is your biggest struggle that you have with daily diabetes care?
- What keeps you on tract?
- What happened when you get off tract?
- How do you manage low blood sugar, what are you thinking and what do you do?
- Has your diabetes affected you relationship with friends and family members?
- Has you diabetes affected you finances?
- Has your diabetes affected you social invitation and how do you cope living with diabetes?

**BIOPHYSICAL PROFILE**

- Height ………………………………………………………….
- Weight…………………………………………………………..
- BMI ……………………………………………………………
- RBS……………………………………………………………..
- Blood pressure…………………………………………………
